# Antitumor Activity of Bioactive Compounds from *Rapana venosa* against Human Breast Cell Lines

**DOI:** 10.3390/ph16020181

**Published:** 2023-01-24

**Authors:** Maria Petrova, Zlatina Vlahova, Maria Schröder, Jordana Todorova, Alexander Tzintzarov, Anastas Gospodinov, Lyudmila Velkova, Dimitar Kaynarov, Aleksandar Dolashki, Pavlina Dolashka, Iva Ugrinova

**Affiliations:** 1 Institute of Molecular Biology “Acad. Roumen Tsanev”, Bulgarian Academy of Sciences, Acad. G. Bonchev Str., Bld. 21, 1113 Sofia, Bulgaria; 2 Institute of Organic Chemistry with Centre of Phytochemistry, Bulgarian Academy of Sciences, Acad. G. Bonchev Str., Bld. 9, 1113 Sofia, Bulgaria

**Keywords:** *Rapana venosa* hemolymph, breast cancer, cisplatin, tamoxifen, bioactive compounds, mass spectrometry

## Abstract

This study is the first report describing the promising antitumor activity of biologically active compounds isolated from the hemolymph of marine snail *Rapana venosa*—a fraction with Mw between 50 and 100 kDa and two structural subunits (RvH1 and RvH2), tested on a panel of human breast cell lines—six lines of different molecular subtypes of breast cancer MDA-MB-231, MDA-MB-468, BT-474, BT-549, SK-BR-3, and MCF-7 and the non-cancerous MCF-10A. The fraction with Mw 50–100 kDa (HRv 50–100) showed good antitumor activity manifested by a significant decrease in cell viability, altered morphology, autophagy, and p53 activation in treated cancer cells. An apparent synergistic effect was observed for the combination of HRv 50–100 with cis-platin for all tested cell lines. The combination of HRv 50–100 with cisplatin and/or tamoxifen is three times more effective compared to treatment with classical chemotherapeutics alone. The main proteins in the active fraction, with Mw at ~50 kDa, ~65 kDa, ~100 kDa, were identified by MALDI-MS, MS/MS analyses, and bioinformatics. Homology was established with known proteins with antitumor potential detected in different mollusc species: peroxidase-like protein, glycoproteins Aplysianin A, L-amino acid oxidase (LAAO), and the functional unit with Mw 50 kDa of RvH. Our study reveals new perspectives for application of HRv 50–100 as an antitumor agent used alone or as a booster in combination with different chemotherapies.

## 1. Introduction

Oncological diseases are characterized by high incidence and mortality and have significant social and health impacts. Cancer is a leading cause of death worldwide, accounting for nearly 10 million deaths in 2020, or nearly one in six deaths [1]. Breast cancer is the most common cancer in women and the second most common cancer overall, with global incidence and mortality rates of 24.2% and 11.6%, respectively, making it the second cause of mortality among women worldwide. Conventional cancer treatments, such as chemotherapy and radiation, have numerous pitfalls, being toxic to healthy cells, leading to many side effects, ineffective induction of cell death, and drug resistance [2]. Monotherapeutic approaches target actively proliferating cells non-selectively, which ultimately leads to the destruction of both healthy and cancerous cells. Chemotherapy can be toxic to patients, with multiple side effects and risks, and strongly compromise their immune system by affecting bone marrow cells [2,3].

A particular case is the treatment of patients diagnosed with triple-negative breast cancer (TNBC) due to high resistance to anticancer drugs. This persuades researchers to look for novel therapeutic approaches to treat this malignancy [4]. One of these approaches is using photosensitizing agents that allow greater therapeutic efficacy and selectivity with fewer side effects [5,6]. Another approach is to look for new compounds isolated from different natural sources, such as plants and invertebrates (land- and water-dwelling); this approach has been of great interest for anticancer research. Some bioactive compounds isolated from the hemolymph of mollusc species exhibit antitumor activity, have antioxidant and immunomodulatory properties, and attract significant interest as good drug candidates for therapeutic applications [7,8,9,10,11].

The compounds in the hemolymph of arthropod and mollusc species with promising anticancer activity are the hemocyanins (Hcs)—oligomeric glycometalloproteins carrying oxygen. They differ primarily in their molecular masses, quaternary structures, carbohydrate content, and composition [12]. Some mollusc hemocyanins have significant immunostimulatory and antitumor potential, including keyhole limpet hemocyanin (KLH) [13,14,15], *Concholepas concholepas* (CCH) [16,17], and *Fissurella latimarginata* (FLH) [17,18]. Clinical studies have shown that KLH treatment significantly reduces tumor recurrence in patients with urinary bladder carcinoma [13,19]. Moreover, KLH significantly reduced the proliferation and viability of pancreatic cancer cells, estrogen-dependent and estrogen-independent breast cancer cells, and Barrett’s esophageal adenocarcinoma cells [20,21]. Our previous studies demonstrated that isoforms and functional units of hemocyanins from *Rapana venosa* (RvH) and *Helix lucorum* (HlH) (previously called *Helix vulgaris* (HvH)) have antitumor activity and immunostimulatory properties [22]. Molluscan hemocyanins are oligomeric glycoproteins with heterogeneous glycosylation patterns, primarily mannose-rich *N*-glycans, which contribute to their structural stability and immunomodulatory properties in mammals. Due to their structural characteristics, molluscan hemocyanins stimulate the immune system of mammals and induce a strong humoral and cellular immune response [18,19,23].

The native RvH is organized into two structural subunits, RvH1 and RvH2, with molecular masses of 420 and 400 kDa, respectively [24,25]. Each structural subunit consists of eight globular functional units (FUs) of approximately 50 kDa with a different carbohydrate content. The carbohydrate component of molluscan hemocyanins has been reported to be up to 9% (*w*/*w*). It contains various sugar moieties, including mannose, D-galactose, fucose, N-acetyl-D-galactosamine, and N-acetyl-glucosamine residues, as well as xylose, which is not usually present in animal proteins [18,25,26,27,28]. Hemocyanins are characterized by numerous *N*-glycosylation sites and limited *O*-glycosylation sites [27,28].

A previous study on the antitumor properties of Hcs demonstrated that RvH, HlH, and *Helix aspersa* hemocyanin (HaH) have an antiproliferative effect on the CAL-29 and T-24 bladder cancer cell lines and the human colorectal carcinoma cell line HT-29 [8,9,10]. The results indicated that these hemocyanins and their isoforms have antineoplastic activity and the potential for developing novel therapeutics.

The present study aims to assess the in vitro antitumor activity of bioactive compounds isolated from the hemolymph of marine snail *R. venosa* against a panel of human breast cell lines that differ in origin and genetic profile, including both non-cancerous (MCF-10A) and cancer cell lines (MDA-MB-231, MDA-MB-468, BT-474, BT-549, SK-BR-3, and MCF-7).

## 2. Results

### 2.1. Isolation of Bioactive Compounds from the Hemolymph of the Marine Snail Rapana venosa

The object of our study is the hemolymph of the Black Sea marine snail *R. venosa,* rich in various bioactive compounds with potential application in medicine. The hemolymph was divided into three fractions (with Mw greater than 100 kDa, between 10 and 100 kDa, and between 50 and 100 kDa) after ultrafiltration using 100 kDa, 50 kDa, and 10 kDa membranes.

**Figure 1:**
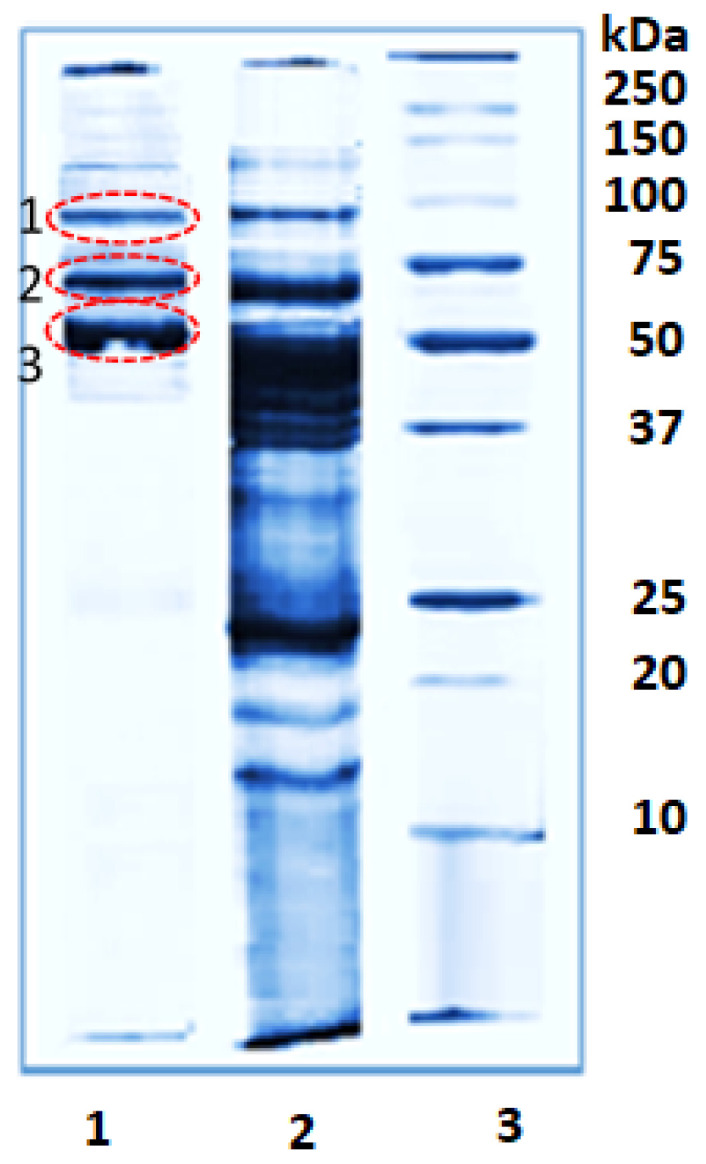
Fractions from *R. venosa* hemolymph analyzed by 12.0% SDS-PAGE visualized by staining with Coomassie G-250: position (1) with Mw 50–100 kDa; (2) with Mw 10–100 kDa; (3) molecular weights of standard proteins from Bio-rad.

The major protein (over 90%) in *R. venosa* hemolymph is hemocyanin (RvH) with a molecular weight of ~8 MDa, isolated from the fraction with Mw greater than 100 kDa after ultracentrifugation at 22,000 rpm and 4 °C for 180 min [10]. RvH is a glycoprotein organized by two structural subunits, RvH1 and RvH2, with a molecular weight of 420 and 400 kDa, respectively. They were isolated after dialysis against 0.13 M glycine/NaOH buffer, pH 9.6 and dissociation of RvH. Other proteins in fractions with Mw below 100 kDa of the hemolymph of *R. venosa* were analyzed by 12.0% SDS-PAGE (Figure 1).

### 2.2. A Glycosylation Screening

**Figure 2:**
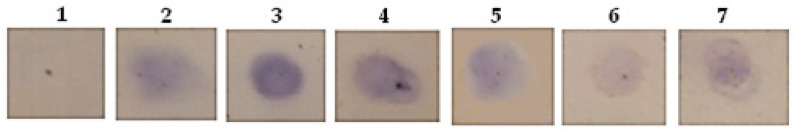
Carbohydrate orcinol/H_2_SO_4_ test. Position 1: negative control (H_2_O); position 2: mannose (0.5 mg/mL); position 3: mannose (1 mg/mL); position 4: RvH1; position 5: RvH2; position 6: fraction with Mw 10–100 kDa; position 7: fraction with Mw 50–100 kDa.

Glycosylation is one of the most common post-translational modifications of proteins and an essential parameter in optimizing many glycoprotein-based drugs, such as monoclonal antibodies [29]. The orcinol-sulfuric acid test showed that all fractions change color except the negative control, water (Figure 2). The test confirmed a higher degree of glycosylation of RvH1 compared to RvH2, as well as of the hemolymph fraction with Mw 50–100 kDa (HRv 50–100 kDa) compared to the fractions with Mw 10–100 kDa.

### 2.3. Identification of Proteins in Active Fraction with Mw 50–100 kDa from R. venosa Hemolymph

The *R. venosa* hemocyanin (RvH), its two structural subunits, as well as their bioactive properties have been extensively investigated [10,22,24,25,26,27,28,30,31,32]. The oligosaccharide structures of its isoforms and some functional units were also characterized [25,26,27,28,32]. Moreover, antimicrobial proline-rich peptides with Mw below 10 kDa isolated from *R. venosa* hemolymph have also been studied [33]. The gene sequence of several functional units of RvH is partially determined, as well as the full sequence of actin in *R. venosa* hemolymph [31,34]. However, other proteins in the hemolymph of *R. venosa* have not yet been studied. Therefore, our study focused on the protein content of the Mw fraction between 50 and 100 kDa, which showed the highest activity against tested breast cancer lines MDA-MB-231 (mutant p53), MCF-7 (wild type p53), and the non-tumorigenic MCF-10A cell line. The results of the SDS-PAGE analysis showed the presence of three major proteins: with Mw ~100, ~65, and ~50 kDa (Figure 1).

**Figure 3:**
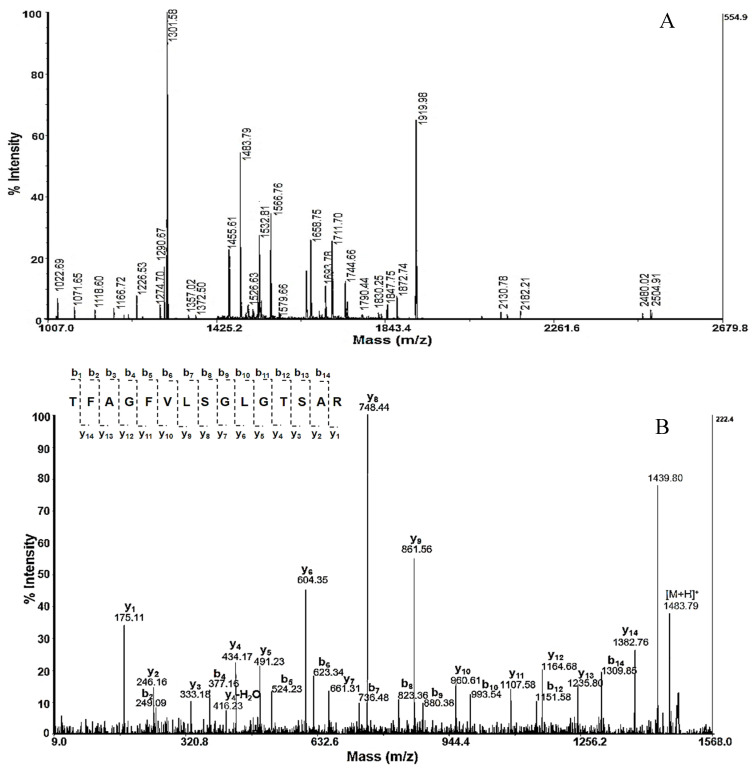
(**A**) MS spectrum of protein on band 2, the fraction with Mw 50–100 kDa of *R. venosa* hemolymph (Figure 1); (**B**) MS/MS spectrum and *de novo* sequence analysis of peptide at *m*/*z* 1483.79 [M+H]^+^.

To identify these proteins, gel bands were excised and digested with trypsin, followed by the extraction of the peptides. The masses of the extracted peptides from each band were determined by MALDI-MS analyses. The peptides obtained by trypsin digestion of the protein in band 2 (a fraction with Mw 50–100 kDa) are presented in Figure 3A.

The amino acid sequences (AASs) of these peptides were determined by MALDI-MS/MS assay. Figure 3B shows the MS/MS analysis of the peptide at *m*/*z* 1483.7903 [M+H]^+^ and the determined AAS after following the y- and b-ions in the spectrum. The same approach was used to determine the AAS of the other peptides obtained after the trypsin digestion of proteins from bands 1, 2, and 3 in Figure 1. The results are presented in Table 1.

The proteins at positions 1, 2, and 3 (Table 1) were identified after aligning the determined AAS with those from the database (by BLAST). The AAS of the protein at position 1 (a fraction with Mw of about 100 kDa) shows high homology with the known proteins peroxidase-like protein 2 and peroxidase-like protein 3 from *Lottia gigantea* (Uniprot ID: B3A0P3, with theoretical Mw 92943 Da), peroxidase-like protein from *Margaritifera margaritifera* (Uniprot ID: H2A0M7), and the peroxidase-like protein from *Mizuhopecten yessoensis* (Uniprot ID: A0A210Q736). We conclude that the protein at position 1 is similar to the peroxidase-like protein family in Mollusca.

For the peptides at position 2 (Figure 3A), homology with two proteins was established in the database. The first protein has good consistency with L-amino acid oxidase (LAAO) from *Aplysia californica* (Uniprot ID: Q6IWZ0, with theoretical Mw 60,300 Da) and Aplysianin A from *Aplysia kurodai* (Uniprot ID: Q17043, with theoretical Mw 62376 Da). The ions at *m*/*z* 1483.83 and *m*/*z* 1693.78 [M+H]^+^ show homology of the second protein with hemocyanins (predominantly functional units) such as RvH2-e, OdH-a to OdH-g, KLH2-c, and the KLH-A subunit.

The protein at position 3 with Mw ~50 kDa was found to be highly homologous to the mollusc hemocyanins (KLH-A, KLH-B, KLH2-c, and RvH2-a) (Table 1). The similarity of the proteins at positions 2 and 3 to the functional units RvH2-a, RvH2-e, OdH-a to OdH-g, KLH2-c let us assume that the proteins in the fraction with Mw between 50 and 100 kDa, identified by 12.0% SDS-PAGE, are functional units of RvH obtained after enzymatic hydrolysis.

### 2.4. Cytotoxic Effect of the Tested Biological Substances Isolated from R. venosa

A comparative study was performed for the first time on the antiproliferative effects of biologically active compounds isolated from the hemolymph and hemocyanin of the marine snail *R. venosa.* This study was conducted on six human breast cancer cell lines and one non-cancerous breast cell line. Three cell lines (MDA-MB-231, MDA-MB-468, and BT-549) have a mutated TP53 gene and express functionally inactive p53 protein. They are a good model for triple-negative, hormone-independent breast cancer, which has a poor prognosis due to its high metastatic potential and drug resistance. Therefore, any substances affecting these subtypes of cancer are of great pharmacological interest. SK-BR-3 cells overexpress human epidermal growth factor receptors 2 (HER-2) but lack expression of estrogen receptors (ER) and progesterone receptors (PR); they also have mutated TP53 gene and functionally inactive p53 protein. BT-474 cells overexpress HER-2, ER, and PR but are characterized by an inactive p53 protein. The MCF-7 cell line is a standard model for a hormone-dependent, less aggressive type of breast cancer; these cells express wild type p53. MCF-10A is widely used as a non-cancerous negative control.

In this study, we used the following biologically active substances (BAS) from *R. venosa* hemolymph: a fraction with Mw between 50 and 100 kDa (HRv 50–100 kDa) and two structural subunits of hemocyanin—RvH1 and RvH2. The widely used chemotherapy drugs cisplatin (cis-Pt) and tamoxifen (Tam), a hormone therapy drug, were used separately as positive controls or in combination with the selected biologically active fractions to investigate potential synergistic effects against the tested tumor cell lines.

Initially, the half-effective dose IC_50_ of the widely used chemotherapy drugs cisplatin and tamoxifen was determined for each cell line. Natural extracts of *R. venosa* were also tested, and the half-effective doses of IC_50_ were determined (Table 2).

As evident in Table 2, the tested natural products are less toxic than cisplatin or tamoxifen, showing higher IC_50_ values. However, one product stood out, *R. venosa* 50–100 kDa hemolymph, with an IC_50_ value in some cases comparable to that of cisplatin, suggesting that the fraction could exhibit antitumor activity on its own.

Furthermore, we focused on two cell lines representing both molecular subtypes of breast cancer—the triple-negative MDA-MB-231 and the hormone-dependent MCF-7.

**Figure 4:**
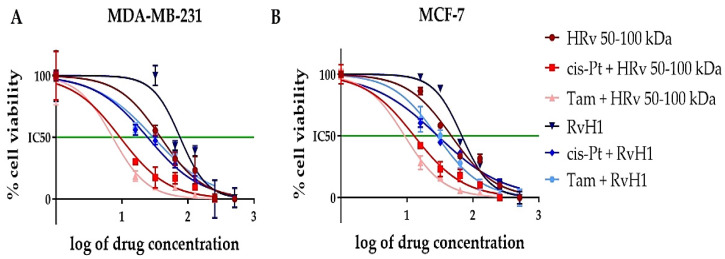
Dose–response curves of cancerous cells treated with HRv 50–100 kDa (dark red line) alone, in combination with cisplatin (bright red line) or tamoxifen (pink line), or RvH1 alone (dark blue line), in combination with cisplatin (bright blue line) or tamoxifen (light blue line). Logarithms of the concentrations of the substances, alone and in combinations, are plotted against cell viability in %. (**A**) MDA-MB-231 treated cells; (**B**) MCF-7 treated cells.

To test a potential synergistic effect, HRv 50–100 kDa at IC_25_ concentrations was combined with cisplatin or tamoxifen applied in serial dilutions from 16 to 512 μM (Table 3). The half-effective doses (calculated from the dose–response curves in Figure 4) were significantly reduced.

Table 3 and Figure 4 showed a serious reduction (around three times) in the half-effective dose for all tested combinations compared to the IC_50_ values of the drugs when applied alone (see Table 2). The most pronounced effect was observed for the combined treatment of the hemolymph fraction of *R. venosa* 50–100 kDa with tamoxifen. These primary results suggest a well-pronounced synergistic effect between known chemotherapeutics cisplatin or tamoxifen and natural products isolated from *R. venosa* and a slight selectivity to cancerous cells. 

We first used the Chou–Talalay method to test the hypothesis for synergism [35]. We used the dose–response data to test the hemolymph fraction from *R. venosa* 50–100 kDa, at a constant concentration corresponding to IC_25,_ in combinations with cisplatin or tamoxifen in serial dilutions from 16 to 512 μM on two breast cancer cell lines MCF-7 and MDA-MB-231. Concentrations were recalculated from μM and presented in µg/mL for all synergistic studies. The obtained data from these MTT analyses were entered in the CompuSyn^®^ software tool, and the combination index (CI) was calculated (Table 4). This approach first detected a synergistic effect for the combined treatment with hemolymph fraction from *R. venosa* 50–100 kDa and cisplatin.

**Figure 5:**
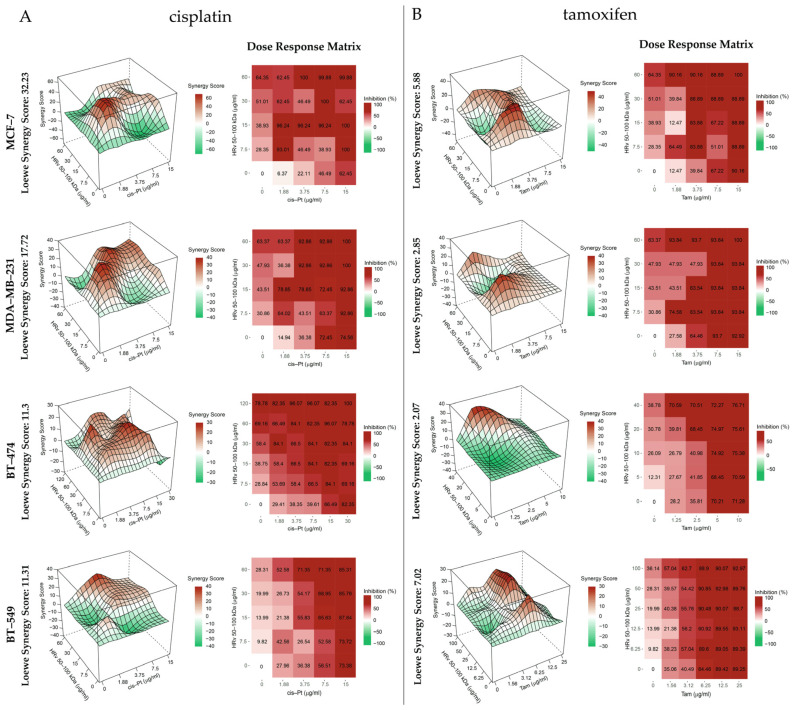
Combination landscapes and dose–response matrices in different cell lines treated with hemolymph fraction of *R. venosa* 50–100 kDa and cisplatin (**A**) or tamoxifen (**B**).

Next, we performed experiments in which the chemotherapeutics (cisplatin or tamoxifen) were mixed with the hemolymph fraction 50–100 kDa and were applied in serial dilutions to the BT-474 and MCF-7 (hormone-dependent) and BT-549 and MDA-MB-231 (triple-negative) cell lines. The synergistic effect was analyzed using the Loewe additivity model in RStudio [36]. The analysis carried out with the SynergyFinderPlus Bioconductor package [37] (Figure 5) indicated a synergistic interaction between the 50–100 kDa fraction and cisplatin in all tested cell lines, the effect being most pronounced in MCF-7. The combined treatment with tamoxifen was mostly additive.

### 2.5. Microscopic Observation and Analysis of the Morphological Changes and Cell Viability Testing by Trypan Blue

**Figure 6:**
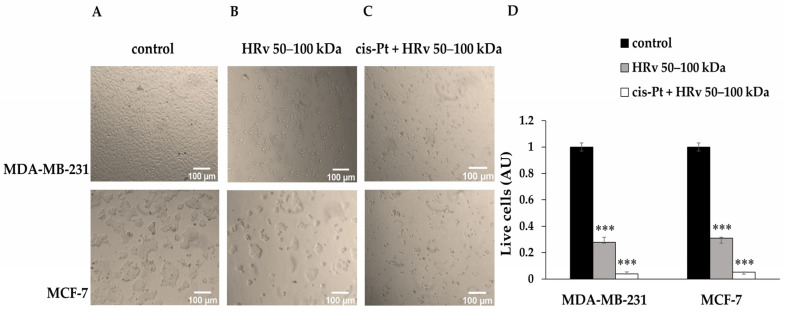
Microscopic images of breast cancer cell lines MDA-MB-231 and MCF-7. (**A**) Control, non-treated cells; (**B**) Cells treated with IC_50_ of hemolymph from *R. venosa* (Mw 50–100 kDa); (**C**) Cells treated with cisplatin at IC_50_ in combination with hemolymph fraction from *R. venosa* with Mw 50–100 kDa at IC_25_; (**D**) Quantification of live cells determined after Trypan Blue staining, calculated as arbitrary units (AU) normalized to the number of live cells in the untreated control. For statistical analysis, Dunnett’s multiple comparisons test in ordinary two-way ANOVA was used to compare the mean of each column with the mean of the control column. Probability values were *** *p* < 0.005, Bar graphs represent the means of four independent experiments.

We performed a microscopic evaluation (Figure 6) of MDA-MB-231 and MCF-7 non-treated cells (Panel A), cells treated with IC_50_ of hemolymph fraction (Mw 50–100 kDa, Panel B), and cells treated with IC_50_ of cisplatin in combination with a constant concentration of the hemolymph fraction equal to IC_25_ concentrations (Panel C).

A total of 72 h after hemolymph fraction treatment, a disturbed cell monolayer, as well as changes in cell morphology, were observed in both cancer cell lines (Figure 6B). The effect was more substantial in the cells treated with the biologically active substance and cisplatin combination. The cells from both cancer lines exhibited characteristic signs of cell death (Figure 6C). To confirm cytotoxicity, we counted live and dead cells after staining with Trypan Blue (Figure 6D).

The number of live cells for each condition was calculated and presented as a fraction of untreated control (Figure 6D). The hemolymph fraction alone reduces the number of viable cells by approximately three times, while combining the same fraction with cisplatin results in more than a 10-fold reduction.

### 2.6. The Effects of the Hemolymph Fraction of R. venosa with Mw 50–100 kDa on the Proliferation of Breast Cancer Cells, Tested by a Colony-Forming Assay

To explore the effect of the fraction with Mw 50–100 kDa from *R. venosa* hemolymph on the proliferation of breast cancer cells, we performed a colony-forming assay in MDA-MB-231 and MCF-7 cells.

**Figure 7:**
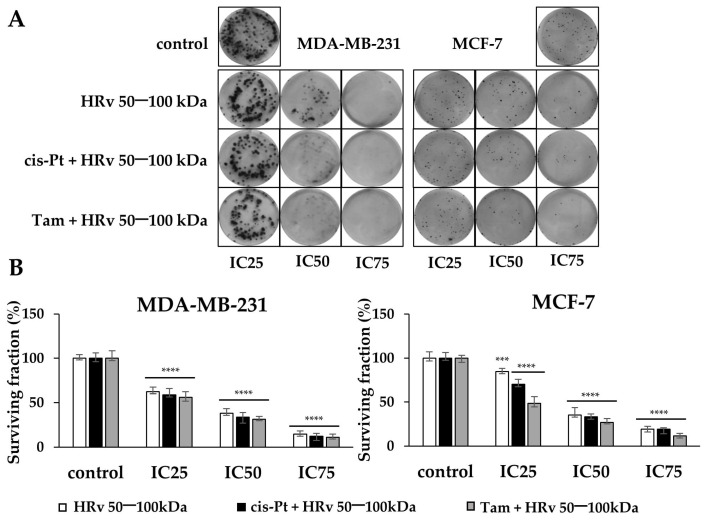
Hemolymph fraction 50-100 kDa and combination treatments with cisplatin (cis-Pt) and tamoxifen (Tam) inhibit colony formation in human breast cancer cells. (**A**) Representative images of colony formation assay conducted in MDA-MB-231 and MCF-7 cells as described in Materials and Methods. Cells were treated with HRv 50–100 kDa and combinations of HRv 50–100 kDa with cisplatin and tamoxifen at indicated IC concentrations for 48 h. After 10 days for MDA-MB-231 and 18 days for MCF-7, the colonies were stained with crystal violet and the number of colonies was counted; (**B**) Quantification of the surviving fraction is represented as the mean ± SD of three independent experiments. For statistical analysis, Dunnett’s multiple comparisons test in ordinary two-way ANOVA was used to compare the mean of each column with the mean of the control column. Probability values were considered significant at *** *p* < 0.005, **** *p* < 0.001. Bar graphs represent the means of three independent experiments.

We also tested the impact of combination treatments of hemolymph fraction 50–100 kDa with cisplatin or tamoxifen on the proliferation ability in both cell lines. As shown in Figure 7, the applied compounds significantly suppressed the capacity to form colonies in both cell lines in a concentration-dependent manner, compared with the matching control group. Interestingly, the triple-negative cancer cells MDA-MB-231 were more sensitive to the antiproliferative activity of the hemolymph from *R. venosa,* as IC_25_ concentrations already reduced their clonogenic survival to 62% and IC_50_ and IC_75_ to 38% and 15%, respectively. MCF-7 cells showed better clonogenic survival after treatment with IC_25_ concentrations of the hemolymph (85%), compared to MDA-MB-231 cells, and a similar dose-dependent reduction in colony formation for IC_50_ and IC_75_. The combined treatment with cisplatin or tamoxifen showed a slightly increased effect compared to the hemolymph fraction alone in both cell lines.

We conclude that the fraction with Mw 50–100 kDa of *R. venosa* hemolymph has a considerable antiproliferative effect on breast cancer cells MDA-MB-231 and MCF-7, affecting their clonogenic potential in a dose-dependent manner.

### 2.7. Immunofluorescent Analysis of p53 Localization and Autophagy Marker LC3 Expression in MCF-7 Cells Treated with Hemolymph from R. venosa 50–100 kDa Alone and in Combination with Cisplatin

In most human cancer cells, the wild type p53 gene is inactivated through mutation, cytoplasmic sequestration, or interaction with negative regulators (MDM2) [38,39,40,41]. Normally, p53 is expressed at low levels and exists in an inactive form in the cytoplasm [42,43]. In response to stress, cytoplasmic p53 is activated and stabilized and accumulates in the nucleus, where it activates gene transcription and triggers growth arrest or apoptosis [44].

Autophagy is an evolutionarily conserved process across eukaryotes and is associated with various pathogenic conditions such as neurodegenerative diseases, inflammation, and cancer. The process can be activated by stress signals and is generally thought to be a survival mechanism, although its deregulation has been linked to non-apoptotic cell death. Typical of this process is the formation of double-membrane structures known as autophagosomes. LC3-II is generated by conjugation of cytosolic LC3-I to phosphatidylethanolamine on the surface of autophagosomes and is observed as LC3-puncta—a marker of autophagy [45].

**Figure 8:**
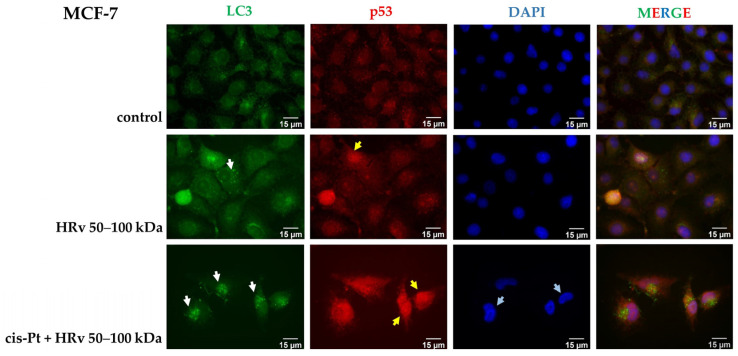
Immunofluorescent analysis of MCF-7 breast cancer control cells (first line), cells treated with hemolymph fraction Mw 50-100 kDa alone at IC_50_ (second line), with cisplatin at IC_50_ in combination with fraction from *R. venosa* at IC_25_ (third line), showing p53 localization (in red) and LC3-puncta formation (in green). Nuclei were stained with DAPI (in blue). The images were taken with a CCD camera AxioCam MRm put on an epifluorescent microscope Zeiss Axiovert 200M.

Immunofluorescent analysis was performed to elucidate the effect of the hemolymph fraction of *R. venosa* 50–100 kDa on p53 activation/re-localization and the initiation of autophagy manifested by LC3 puncta formation. In these experiments, only the MCF-7 cancer cell line (which expresses functional p53 protein) was analyzed. MCF-7 cells were treated with IC_50_ of the hemolymph fraction 50–100 kDa alone or with cisplatin (IC_50_) in combination with the hemolymph fraction of *R. venosa* 50–100 kDa at IC_25_ for 48 h. In control cells, p53 is localized predominantly in the cytoplasm and, corresponding to the usual physiological level of autophagy, a low number of LC3-positive structures are formed (Figure 8, first line). After hemolymph treatment, a clear increase in the number of LC3-puncta (Figure 8, white arrows) and the nuclear import of p53 were observed (Figure 8, yellow arrows), the shape of the nuclei changed (they appear more spindle-like), and bright areas of condensed chromatin appeared (Figure 8, blue arrows). LC3-puncta formations, as well as p53 re-localization in the nucleus, were clearly visible after the treatment with hemolymph from *R. venosa* in combination with cisplatin.

These observations are in line with the evidence that p53 activates autophagy by inducing the expression of genes related to the autophagy [46]. Furthermore, excessive or uncontrolled levels of autophagy can induce an autophagy-dependent cell death [47], which can be associated with the decreased cell viability in treated cells.

## 3. Discussion

Molluscan hemocyanins are widely used in many immunological and clinical applications as natural, non-toxic, non-pathogenic, and nonspecific immunostimulants [8,9,10,13,17,18,19,20,22,23,48,49,50,51,52]. The various biological activities of KLH, CCH, and FLH motivated researchers to study new undescribed hemocyanins for antitumor properties.

The first communication that KLH directly inhibits the growth of human breast and pancreas cancer cells in vitro came 15 years ago from Riggs and co-authors, showing that the cells died by both apoptotic and nonapoptotic mechanisms [21]. In the present study, the antitumor activity of subunits of *R. venosa* hemocyanin (RvH1 and RvH2) and a fraction from *R. venosa* hemolymph was examined on a panel of human breast cancer cell lines: six lines of different molecular subtypes of breast cancer MDA-MB-231, MDA-MB-468, BT-474, BT-549, SK-BR-3, and MCF-7 and the non-cancerous MCF-10A.

Our comparative study of the antiproliferative effects of the biologically active compounds of the hemolymph isolated from the marine snail *R. venosa* on the tested cell lines showed that a fraction with Mw 50–100 kDa has a significant cytotoxic effect on the cancer cells, followed by the effect of the RvH1 structural subunit. These results are consistent with published data on the cytotoxic and/or proapoptotic activity of the RvH subunits in various tumor cell lines [8,9,10,13,15,50,51,52]. The change in the protein glycosylation pattern is linked to malignant transformation and neoplastic progression. Commonly, biologically active molecules with anticancer activities are reported to be glycosylated [53,54,55]. In our study, we found that the RvH1 subunit and the Mw 50–100 fraction are glycosylated.

It can be speculated that the tumor inhibitory activity of RvH and its structural subunits is due to specific oligosaccharide residues [25,26,27,28]. The observed antiproliferative effect of the fraction with Mw between 50 and 100 kDa of the *R. venosa* hemolymph is probably due to a synergistic action of proteins with Mw about 100 kDa, 65 kDa, and 50 kDa (positions 1, 2, and 3, Figure 1). Our analyses have shown that these proteins are homologous to peroxidase-like protein, glycoproteins Aplysianin A and L-amino acid oxidase (LAAO), and the functional units of molluscan hemocyanins KLH, RvH, and OdH, each of which is characterized by prominent antitumor properties [55,56,57,58,59]. *N*-glycans are characteristic of gastropods and may be responsible for the antitumor properties of the biologically active fraction with Mw 50–100 kDa [59].

In this study, we demonstrate that biologically active substances isolated from the marine snail *R. venosa* have antitumor activity against both triple-negative MDA-MB-231, MDA-MB-468, BT-549 and hormone-sensitive MCF-7, BT-474, SK-BR-3 breast cancer cell lines. At the same time, they are not that toxic for the non-cancerous MCF-10A cells, which indicate some selectivity. It is worth stressing that the active compounds, hemolymph from *R. venosa* with Mw 50–100 kDa and isoform of *R. venosa* hemocyanin (RvH1), demonstrate a good synergistic effect in combination with classical chemotherapeutic drugs widely used in oncology—cisplatin and tamoxifen. Tamoxifen is the oldest, most well-known, and most widely prescribed selective estrogen receptor modulator (SERM) commonly used for hormonal therapy in breast cancer, which, unfortunately, is ineffective against TNBC. This kind of malignancy is characterized by high invasive potential and resistance to hormone therapy.

Analyses of the combined effect of 50–100 kDa hemolymph fraction with cisplatin or tamoxifen indicated synergistic interaction with the former drug and additive effect with the latter. This is to be expected, given: (1) the different modes of action of cisplatin and tamoxifen and (2) the applied Loewe model which assumes similar modes of action of the interacting drugs when testing for synergism. Obtained results suggest that the fraction with Mw 50–100 kDa from R. venosa hemolymph also mediates apoptosis, similar to cisplatin. In support of such a hypothesis are the results of the colony formation assay, showing that the 50–100 kDa hemolymph fraction inhibits the proliferation of treated cells close to the extent of cisplatin (Figure 7). We hypothesis the pathway of mediated apoptosis from HRv 50–100 kDa is different than the cisplatin pathway, because the structural characteristics and bioactive properties of glycosylated proteins in this hemolymph fraction and cisplatin are completely different. It is known that basic pharmacological mechanism of cisplatin involves internal and interchain crosslinks created by binding to DNA, inhibition of DNA replication and transcription, and then induction of damage to double-stranded DNA, i.e., apoptosis as a DNA damage response [60]. It is possible that glycoproteins in the HRv 50–100 kDa fraction (which are homologous to peroxidase-like protein, glycoproteins Aplysianin A, L-amino acid oxidase (LAAO), and the functional unit with Mw 50 kDa from RvH) are involved in the induction of cell death in different ways, due to their specific oligosaccharide structures, similarly to molluscan Hcs. Therefore, glycosylation may be an important part of the interaction of these molecules and target tumor cells. It is known that antitumor activity of gastropod hemocyanins might be the consequence of their interactions with glycoproteins located at the membrane of the tumor cells that induce cross-reactive antibodies and promote cellular cytotoxicity [8,9,10,50,51,52,61,62]. Several studies showed that the treatment of different tumor cell lines with various forms of Hcs (for example RvH, HaH, HlH, *L. vannamei* Hc) leads to significant changes in the expression of multiple proteins; more of them play a role in the glycolytic pathway, cellular energy metabolism, cell signal transduction, cell proliferation, and apoptosis, as well as the lysosomal, and proteasome degradation pathways [9,52,63]. In this way, some Hcs mediate antiproliferative properties through the apoptosis mechanism involving the mitochondria-triggered pathway [63]. Therefore, we suggest that antitumor activity of HRv 50–100 kDa could be related to changes in the expression of key proteins, involved in the metabolic pathways of the apoptotic induction of cell death.

It is worth mentioning here that we observed a good synergistic score for combined treatment with *R. venosa* with Mw 50–100 kDa and cisplatin of two cell lines MDA-MB-231 and BT-549, which are a good model for triple-negative, hormone-independent breast cancer. Our observations reveal new possibilities for the combined application of natural, non-toxic biological active compounds together with classical drugs to increase the antitumor activity and decrease the general toxicity of chemotherapeutic drugs.

## 4. Materials and Methods

### 4.1. Cell Cultures

All of the cell lines were obtained from ATCC, LGC Standards and cultivated in conditions of 5% CO_2_ at 37 °C. MCF-7 cells were grown in Eagle’s Minimum Essential Medium (Thermo Fisher Scientific, Watham, MA, USA) with 0.01 mg/mL human recombinant insulin (Sigma-Aldrich, St. Louis, MO, USA). BT-474 cells were cultured in ATCC Hybri-Care Medium 46-X (Thermo Fisher Scientific). SK-BR-3 cells were cultured in McCoy’s 5a Medium Modified (Thermo Fisher Scientific). The base medium for the MDA-MB-468 and MDA-MB-231 cell lines is Leibovitz’s L-15 Medium (Thermo Fisher Scientific). BT-549 cells were cultivated in RPMI 1640 Medium (Thermo Fisher Scientific) with 0.023 U/mL insulin (Sigma-Aldrich).

The immortalized MCF-10A cell line was grown in DMEM-F12 (Thermo Fisher) media supplemented with hydrocortisone 0.5 mg/mL, insulin 10 mg/mL, hEGF 20 ng/mL, 100 ng/mL cholera toxin (all from Sigma-Aldrich).

To all basic growth media, 10% fetal bovine serum (Thermo Fisher Scientific) and penicillin-streptomycin (Thermo Fisher Scientific) were added.

### 4.2. Isolation of Bioactive Compounds from the Hemolymph of Marine Snail R. venosa

The *R. venosa* hemolymph was collected from marine snails living in the Black Sea after cutting the foot muscles. After collection, the hemolymph was filtrated and centrifuged at 10,000 rpm and 4 °C for 20 min to remove rough particles and hemocytes, and the native crude hemolymph was purified. The hemolymph supernatant was divided into two main fractions containing compounds with Mw below and above 100 kDa by ultra-filtration via a membrane filter (Millipore™ 100 kDa Ultrafiltration Membrane Filters, Regenerated cellulose).

#### 4.2.1. Isolation of *R. venosa* Hemocyanin and Its Structural Subunits

The fraction above 100 kDa from *R. venosa* hemolymph, which contains mostly he-mocyanin, was subjected to ultracentrifugation at 22,000 rpm and 4 °C for 5 h with a rotor Kontron-Hermle A8.24, (centrifuge CENTRIKON). After removal of the supernatant, the sediment containing the native *R. venosa* hemocyanin (RvH) was solubilized at a concentration of about 10% in 50 mM Tris buffer (pH 7.5) containing 20 mM CaCl_2_ and 10 mM MgCl_2_. Dissociation of native RvH was achieved by dialyzing the protein against 0.13 M glycine/NaOH buffer, pH 9.6. The structural subunits RvH1 and RvH2 were separated by ion-exchange chromatography on the Sepharose High Performance column by the FPLC system. The column was equilibrated with 50 mM Tris/HCl buffer containing 10 mM EDTA, pH 8.5, with a linear gradient of 0.0–0.5 M NaCl as described previously [10].

#### 4.2.2. Isolation of Bioactive Fraction from Hemolymph of *R. venosa*

A fraction with Mw 50–100 kDa from *R. venosa* hemolymph was obtained via additional ultrafiltration of the fraction below 100 kDa on a 50 kDa membrane Millipore™ (Ultrafiltration Membrane Filters, Regenerated cellulose with a cutoff 50K MWCO).

### 4.3. SDS-PAGE Electrophoresis

Protein fractions from *R. venosa* hemolymph were analyzed by sodium dodecyl sul-fate-polyacrylamide gel electrophoresis (SDS-PAGE) with the molecular weight marker ranging from 250 kDa to 10 kDa using a 5% stacking gel and 12.0% resolving gel, according to the Laemmli method with modifications [64]. DL-dithiothreitol, acrylamide/bis-acrylamide (30% solution), bromophenol blue sodium salt (Sigma-Aldrich, Schnelldorf, Germany), N, N, N′, N′-tetramethylethylenediamine (TEMED), ammonium persulphate (APS) (GE Healthcare, Stockholm, Sweden), and Laemmli sample buffer (2×), for SDS PAGE (SERVA, Heidelberg, Germany), were used for electrophoresis analysis. Equal volumes containing approximately 20 μg/lane of the samples dissolved in Laemmli sample buffer and protein standard mixture (Precision Plus Protein™, All Blue, Bio-Rad, Feldkirchen, Germany) were applied on 12.0% SDS-PAGE and visualized by staining with Coomassie Brilliant Blue G-250 (Bio-Rad Laboratories GmbH, Germany).

### 4.4. A Glycosylation Screening

Fractions isolated from the hemolymph of *R. venosa* were analyzed with the orcinol-sulphuric test to determine their carbohydrate content. About 2 μL of the purified samples were applied to a thin layer plate and air-dried. The plate was sprayed with orcinol/H_2_SO_4_ and heated for 20 min at 100 °C. The orcinol/H_2_SO_4_ solution contained 0.02 g of orcinol, 20% H_2_SO_4_, and H_2_O to a total volume of 10 mL.

### 4.5. Analyses of Proteins after Tryptic Digestion

The protease digestion was run according to the work of Rosenfeld et al. with a slight modification [65]. The target protein bands excised from the SDS-PAGE gels were washed twice with 150 µL mixture of 50% acetonitrile (ACN) and 200 mM NH_4_HCO_3_ each for 20 min at 30 °C to decolorize. The digestion of proteins in gel was carried out with porcine trypsin (Promega, Madison, WI, USA). After drying the decolorized gels in the speedvac concentrator for 30 min, a volume of 10 μL digestion buffer (50 mM ammonium bicarbonate, pH 7.8, containing modified trypsin) was added to them, and the Eppendorf tubes were kept on ice for 45 min to allow the gel pieces to be completely soaked with the protease solution. Digestion was performed overnight at 37 °C, the supernatants were recovered, and the resulting peptides were extracted twice with 35 μL of 60% ACN/0.1% HCOOH. The extracts were pooled and dried in the speedvac concentrator. The extracted peptides were re-dissolved in 10 μL of 0.1% formic acid.

### 4.6. Mass Spectrometry Analysis

The peptides extracted from the gel were analyzed by mass spectrometry (MS- and MS/MS- analyses) on AutoflexTM III, High Performance MALDI-TOF & TOF/TOF Systems (Bruker Daltonics, Bremen, Germany) which uses a 200 Hz frequency-tripled Nd–YAG laser operating at a wavelength of 355 nm. Analysis was carried out using α-cyano-4-hydroxycinnamic acid (CHCA) as a matrix. A total of 2.0 μL of the sample was mixed with 2.0 μL of matrix solution (7 mg/mL of CHCA) in 50% CN containing 0.1% TFA, and only 1.0 μL of the mixture was spotted on a stainless steel 192-well target plate. The samples were dried at room temperature (RT) and subjected to mass analysis. A total of 3500 shots were acquired in the MS mode, and collision energy of 4200 was applied. The mass spectrometer was externally calibrated with a mixture of angiotensin I (1296.6848 Da), angiotensin II (1046.5418 Da), glu-fibrinopeptide B (1569.65 Da), ACTH (1–17) B (1569.65 Da), and ACTH (18–39) (2465.1983 Da). The instrument was externally calibrated with fragments of Glu-fibrinopeptide B for MS/MS experiments. Database SwissProt and NCBI BLAST were done with the amino acid sequences revealed by manual interpretation of the MS/MS spectra.

### 4.7. Cytotoxic Assay

The cell viability was assessed using the standard MTT-dye reduction assay as de-scribed previously [66] with some modifications [67,68]. The method is based on the bi-otransformation of the yellow tetrazolium salt MTT (3-(4,5-Dimethylthiazol-2-yl)- 2,5-diphenyltetrazolium bromide) to a violet formazan via the mitochondrial succinate dehydrogenase in viable cells. Briefly: the exponentially growing cells were seeded in 96-well flat-bottomed microplates (Corning Costar Flat Bottom Cell Culture Plate, Corning, New York, NY, USA) at a density of 2 × 10^3^ cells per well. After 24 h incubation at 37 °C, the cells from each cell line were treated with the corresponding biologically active substances alone, in concentrations ranging from 16 to 512 µg/mL, or in combination with the chemotherapy drug cisplatin (Sigma-Aldrich) or the hormone therapy drug tamoxifen (Sigma-Aldrich) for 72 h (see Table 2). At least 8 wells were used for each concentration. In some experiments, the cells from the different cell lines were treated with a combination of a constant concentration of the biologically active substances isolated from *R. venosa* (corresponding to the IC_25_ values as shown in Table 3) and serial dilutions of cisplatin or tamoxifen ranging from 16 to 512 µM. In other experiments, the chemotherapeutics (cisplatin or tamoxifen) were mixed with the hemolymph fraction 50–100 kDa and were applied in serial dilutions together (see Figure 5). Cisplatin and tamoxifen were dissolved in DMSO. The maximal final concentration of DMSO in the treating solutions was 2.5%. After a 72 h incubation in 5% CO_2_ at 37 °C, the medium was changed with a phenol-red-free medium, and MTT (Invitrogen) was added in a final concentration of 0.5 mg/mL. The cells were incubated for 2 h in 5% CO_2_ at 37 °C. Finally, 100µL DMSO per well was added to dissolve the formed formazan crystals. The measurement of the absorbance of the samples was performed on a Varioskan LUX Multimode Microplate Reader (Thermo Fisher Scientific) at 570 nm. GraphPad Prism software v.8 was used for data analysis.

### 4.8. Colony Formation Assay

The in vitro colony formation assay was used to evaluate cell survival and proliferation based on the ability of a single cell to grow into a colony. The colony was defined to consist of at least 50 cells. The cells from MDA-MB-231 and MCF-7 were seeded in 6-well flat-bottomed plates (Corning Costar Flat Bottom Cell Culture Plate) at 200 or 300 cells per well, respectively, at a single-cell density—without any clumps of cells present. After 24 h of incubation at 37 °C, the cells from the two cancer cell lines were treated for 48 h with concentrations corresponding to the IC_25_, IC_50_, and IC_75_ values of the hemolymph fraction of *R. venosa* with Mw 50–100 kDa, the combination of cisplatin with hemolymph 50–100 kDa, and the combination of tamoxifen with the same fraction. The medium was then replaced with fresh medium, and the cells were incubated for 10 days (MDA-MB-231) or 18 days (MCF-7) at 5% CO_2_ and 37 °C. After incubation, the cells were fixed with 4% PFA for 20 min at gentle agitation. Then, the cells were rinsed with 1 × PBS for 5 min and stained with 0.2% Crystal-Violet (Sigma-Aldrich) for 20 min at room temperature at gentle agitation. After staining, the cells were washed 3 × 5 min with dH_2_O and dried at room temperature. Then, the colonies were counted on Image software, and plating efficiency (PE) and survival fraction (SF) were calculated as described [69]. The images were processed by Photoshop^®^ V. 13 CS6 software, and data were analyzed with statistics software for graphical representation.

### 4.9. Microscopic Observations

Viability was also determined upon visual inspection via phase contrast microscopy and Trypan Blue staining [70]. After staining for 5 min with 0.4% Trypan Blue solution (Thermo Fisher Scientific), the cells were counted in a Cell Counter (Corning, New York, NY, USA) chamber, and the percentage of live cells was determined.

### 4.10. Immunofluorescent Microscopy 

Cells from the breast cancer cell line MCF-7 were seeded in numbers of 3 × 10^4^ per well in a 24-well plate (Corning Costar Flat Bottom Cell Culture Plate) on glass coverslips. After 24 h, the cells were treated with concentrations corresponding to the IC_50_ values (calculated with GraphPad Prism V.7) of hemolymph of R. venosa with Mw 50–100 kDa alone and in combination with cisplatin. Immunofluorescence analysis was performed as previously described [70]. For p53 and LC3 detection, mouse monoclonal (Biologend, San Diego, CA, USA) antibody in 1:50 and rabbit polyclonal (Abcam) antibody in 1:2000 dilutions were used, respectively. The signal was visualized with secondary Donkey anti-mouse Alexa Fluor 555 (Thermo Fisher Scientific, Watham, MA, USA) and Donkey anti-rabbit Alexa Fluor 488 (Thermo Fisher Scientific, Watham, MA, USA) antibodies, both at 1:2000 dilution. The glass coverslips were mounted in ProLong Diamond Antifade Mountant (Thermo Fisher Scientific, Watham, MA, USA) containing 10 μg/mL DAPI. The signals were obtained on a Zeiss AxioVert 200M microscope using a 63× objective lens, equipped with a CCD camera AxioCam MRm. The images were taken under the same settings and processed with ImageJ.

### 4.11. Chou–Talalay Method for Determination of the Combination Index (CI)

The synergistic, antagonistic, or additive cytotoxic effects of the drug (tamoxifen or cisplatin) combination with fraction from *R. venosa* hemolymph with Mw 50–100 kDa were determined with the Chou–Talalay method [35]. To analyze the cytotoxicity of combination treatments, we carried out MTT-dye reduction assay (as described above), applying a non-constant ratio of the compounds. We used a fixed concentration of the hemolymph fraction corresponding to the IC_25_ value for each cancer cell line and concentrations of tamoxifen or cisplatin ranging from 1.5 to 190 µg/mL and 1.2 to 154 µg/mL, equivalent to the concentrations in µM, respectively. The dose–response data were entered in the CompuSyn^®^ software tool, and the combination index (CI) was calculated. 

The synergy scores for each pair of drug combinations used to treat the panel of cells were calculated using the Bioconductor package SynergyFinderPlus [37] using the Loewe model in RStudio.

## 5. Conclusions

The discovery of more effective and selective antitumor medicines with natural origin is one of the main trends of contemporary oncology research. In the current study, we showed that hemolymph fraction from marine snail *R. venosa* 50–100 kDa has a promising antitumor activity manifested by a significant decrease in the cell viability, disturbed morphology, and activation of autophagy. This active fraction showed slight selectivity being less toxic to the non-cancerous MCF-10A cell line. Of potential importance is the finding that the studied hemolymph fraction demonstrates significant synergistic and additive effects in combination with classical chemotherapeutic drugs widely used in oncology—cisplatin and tamoxifen, respectively. Surprisingly, our results revealed that cisplatin applied in combination with hemolymph from *R. venosa* with Mw 50–100 kDa is about three times more active against TNBC compared to cisplatin alone.

Our study reveals new perspectives for the application of the studied hemolymph fraction as an antitumor agent used alone or as an adjuvant to different chemotherapies.

## Figures and Tables

**Table 1:** Amino acid sequences (AASs) of peptides, determined after analysis of their MS/MS spectra. Proteins were identified after comparing AASs with database of protein sequences by the Basic Local Alignment Search Tool (BLAST).

Band	AAS of Peptide	Mass Exp. [M+H]^+^	Protein Name	UniProt ID	Identities
**1**	HGDDCCDMDMR	1297.41	Peroxidase-like protein 2 [*L. gigantea*]	B3A0P3	100%, E = 0.20
	DHGEPPYDDFR	1347.56	Peroxidase-like protein 2 [*L. gigantea*]	B3A0P3	73%, E = 0.035
	LPGAFTGPTFNCIAR	1635.83	Peroxidase-like protein 3 [*L. gigantea*]	B3A0Q8	63%, E = 0.001
			Peroxidase-like protein 2 [*L. gigantea*]	B3A0P3	63%, E = 0.015
			Peroxidase-like protein [*P. margaritifera*]	H2A0M7	67%, E = 0.12
**2.1**	MPAQPVAGLFDR	1301.58	Peroxidase-like protein 2 [*L. gigantea*]	B3A0P3	100%, E = 0.021
	LDWPVLFNDR	1274.7	Aplysianin-A [*Aplysia kurodai*]	Q17043	83%, E = 0.97
	KLFWHMDWK	1290.67	L-amino-acid oxidase LAAO [*A. califonica*]	Q6IWZ0	63%, E = 1.0
	MFHFDELLDLPR	1532.81	L-amino-acid oxidase LAAO [*A. californica*]	Q6IWZ0	86%, E = 0.35
			Aplysianin-A [*A. kurodai*]	Q17043	86%, E = 0.35
	DYHFDELLDLMR	1566.76	Aplysianin-A [*A. kurodai*]	Q17043	55%, E = 0.085
			L-amino-acid oxidase LAAO [*A. californica*]	Q6IWZ0	55%, E = 0.17
	YDRWDVPEPEFVVLR	1919.98	Aplysianin-A [*A. kurodai*]	Q17043	63%, E = 14
**2.2**	TFAGFVLSGLGTSAR	1483.79	Hemocyanin type 2 unit-e; RvH2-e [*R. venosa*];	P83040	77%, E = 5e-04
			Hemocyanin G-type, units OdHa-g [*E.dofleini*]	O61363	77%, E = 4e-04
	EYRYYWDWQER	1693.78	Hemocyanin 1; Keyhole limpet hemocyanin A (KLH-A) [*M. crenulata*]	Q10583	83%, E = 0.034
			Hemocyanin G-type, units OdHa-g [*E.dofleini*]		
			Hemocyanin, units g and h [*Sepia officinalis*]	O61363	100%, E = 0.099
			Hemocyanin 2-c chain; KLH2-c [*M. crenulata*]	P56826	100%, E = 0.100
				P81732	100%, E = 0.100
**3**	GHKKRLRK	1022.68	Hemocyanin 2-c chain; KLH2-c [*M. crenulata*],	P81732	63%, E = 3.7
	DEVVPNPFVR	1171.61	Hemocyanin 1; KLH-A [*M. crenulata*]	Q10583	75%, E = 0.057
			Hemocyanin type 2 unit a; RvH2-a [*R. venosa*]	P80960	100%, E = 0.33
			Hemocyanin 2; KLH-B [*M. crenulata*]	Q10584	75%, E = 0.48
	VEITKALHKLGLR	1477.92	Hemocyanin 2-c, KLH2-c [*M. crenulata*]	P81732	64%, E = 0.29
	YHRQEHRRWWKD	1796.9	Hemocyanin 1; KLH-A [*M. crenulata*]	Q10583	83%, E = 1.0

**Table 2:** IC_50_ of BAS, isolated from *R. venosa*, tested on six breast cancer cell lines and the non-cancerous MCF-10A breast cell line. Widely used chemotherapy drugs cisplatin (cis-Pt) and tamoxifen (Tam) were used as positive controls.

BAS/Cells	MCF-10A	MCF-7	BT-474	SK-BR-3	MDA-MB-468	BT-549	MDA-MB-231
cis-Pt [µM]	28 ± 1.8	19.6±2.5	19.3 ± 4.4	20.5 ± 2.1	3 ± 3.3	13.1±3.7	24 ± 3.1
Tam [µM]	26 ± 2.6	18±3.4	2.9 ± 4.4	9.3 ± 1.2	13.8 ± 4.1	9.8 ± 2.2	21 ± 2.4
RvH1 [μg/mL]	68 ± 3.9	63±4.1	67 ± 3.2	96 ± 2.3	79 ± 2.6	92 ± 3.6	72 ± 4.9
RvH2 [μg/mL]	204 ± 4.7	198±3.7	207 ± 3.4	210 ± 1.7	213 ± 3.7	218± 1.8	215 ± 5.1
HRv 50–100kDa [μg/mL]	47 ± 2.8	32 ±3.3	26.2 ± 2.7	74.5 ± 2.5	41.2 ± 4.6	80.4±4.1	38 ± 2.9

**Table 3:** IC_25_ of BAS, isolated from *R. venosa*, in combinations with cisplatin or tamoxifen in serial dilutions from 16 to 512 μM tested on breast cancer cell lines MCF-7 and MDA-MB-231 and the non-cancerous MCF-10A breast cell line.

BAS/Cells	MCF-10A	MCF-7	MDA-MB-231
cis-Pt [μM]+ RvH1 [IC25 = 23 μg/mL]	29 ± 3.4	22 ± 4.3	24 ± 3.5
cis-Pt [μM] + RvH2 [IC25 = 70 μg/mL]	28 ± 2.8	32 ± 3.6	26 ± 3.5
cis-Pt [μM] + HRv 50–100 kDa [IC25 = 13 μg/mL]	13 ± 2.6	10 ± 3.3	9 ± 2.1
Tam [μM]+ RvH1 [IC25 = 23 μg/mL]	28 ± 3.7	25 ± 2.4	33 ± 1.8
Tam [μM] + RvH2 [IC25 = 70 μg/mL]	42 ± 3.8	48 ± 5.2	30 ± 4.4
Tam [μM] + HRv 50–100 kDa [IC25 = 13 μg/mL]	9 ± 2.4	6 ± 1.6	7 ± 2.2

**Table 4:** Parameters of the combination index analysis in CompuSyn^®^ for MCF-7 and MDA-MB-231 cells treated with hemolymph fraction from *R. venosa* with Mw 50–100 kDa at a constant concentration corresponding to IC_25_ combined with cisplatin or tamoxifen in serial dilutions from 16 to 512 μM. The graded symbols for column “Combined effect” are designated as: Antagonism (1.45–3.3) = ---; Moderate antagonism (1.20–1.45) = --; Slight antagonism (1.10–1.20) = -; Nearly additive (0.90–1.10); Slight synergism (0.85–0.90) = +; Moderate synergism (0.7–0.85) = ++; Synergism (0.3–0.7) = +++; Strong synergism (0.1–0.3) = ++++.

**MDA-MB-231**									
**Dose HRv 50** **–100 kDa (µg/mL)**	**Dose cis** **-Pt (µg/mL)**	**Fa**	**CI**	**Combined Effect**	**Dose HRv 50** **–100 kDa (µg/mL)**	**Dose Tam (µg/mL)**	**Fa**	**CI**	**Combined Effect**
12	1.5	0.30	0.92	**±**	12	1.2	0.06	2.16	**---**
12	3	0.48	0.86	**+**	12	2.4	0.26	1.32	**--**
12	6	0.61	0.84	**++**	12	4.8	0.70	0.83	**++**
12	12	0.5	0.88	**+**	12	9.8	0.83	1	**±**
12	24	0.86	1.67	**---**	12	19.2	0.83	1.77	**---**
12	48	0.96	1.72	**---**	12	38	0.90	2.59	**---**
12	95	1.00	0.62	**+++**	12	77	1.00	0.59	**+++**
**MCF-7**									
**Dose HRv 50** **–100 kDa (µg/mL)**	**Dose cis-Pt (µg/mL)**	**Fa**	**CI**	**Combined Effect**	**Dose HRv 50** **–100 kDa (µg/mL)**	**Dose Tam (µg/mL)**	**Fa**	**CI**	**Combined Effect**
12	1.5	0.16	2.13	**---**	12	1.2	0.26	1.36	**--**
12	3	0.59	0.79	**++**	12	2.4	0.44	0.95	**±**
12	6	0.79	0.69	**+++**	12	4.8	0.60	0.83	**++**
12	12	0.98	0.33	**++++**	12	9.8	0.74	0.84	**++**
12	24	0.99	0.39	**+++**	12	19.2	0.80	1.12	**-**
12	48	1.00	0.38	**+++**	12	38	0.84	1.74	**---**
12	95	1.00	0.71	**++**	12	77	0.96	1.09	**-**
12	190	1.00	0.93	**++**	12	154	1.00	0.15	**++++**

## Data Availability

Data is contained within the article.
